# Low tumour burden is associated with observation after surgery in patients with grade 2 astrocytoma and oligodendroglioma: results from the prospective multicentre LoG-Glio registry

**DOI:** 10.1007/s11060-025-05279-4

**Published:** 2025-10-15

**Authors:** Andreas Ziebart, Julia Onken, Minou Nadji-Ohl, Christine Jungk, Stefan Rückriegel, Dorothee Mielke, Rüdiger Gerlach, Marie-Therese Forster, Constantin Roder, Katja Kniese, Nicolas Neidert, Ludovica Fabbrocini, Darius Kalasauskas, Gerges Aziz, Sabrina Riehl, Lennart Sannwald, Benjamin Mayer, Christian Rainer Wirtz, Daniel Sachs, Sebastian Ille, Mario Löhr, Rainer Ritz, Florian Ringel, Florian Ebner, Roland Roelz, Jürgen Beck, Arya Nabavi, Marcos Tatagiba, Marcus Czabanka, Veit Rohde, Ralf-Ingo Ernestus, Sandro Krieg, Oliver Ganslandt, Peter Vajkoczy, Jan Coburger

**Affiliations:** 1 Department of Neurosurgery, Ulm University Hospital, 89081 Ulm, Germany; 2 Department of Neurosurgery, Charité Universitätsmedizin Berlin, 12200 Berlin, Germany; 3 Department of Neurosurgery, Klinikum Stuttgart, 70174 Stuttgart, Germany; 4 Department of Neurosurgery, Medical Faculty, Heidelberg University, 69120 Heidelberg, Germany; 5 Department of Neurosurgery, University of Würzburg, 97080 Würzburg, Germany; 6 Department of Neurosurgery, University of Göttingen, 37075 Göttingen, Germany; 7 Department of Neurosurgery, University Hospital Augsburg, 86156 Augsburg, Germany; 8 Department of Neurosurgery, Helioskliniken Erfurt, 99089 Erfurt, Germany; 9 Department of Neurosurgery, University of Frankfurt, 60528 Frankfurt am Main, Germany; 10 Department of Neurosurgery, University of Tübingen, 72076 Tübingen, Germany; 11 Department of Neurosurgery, Klinikum Region Hannover GmbH, Hannover, Germany; 12 Department of Neurosurgery, Medical Centre - University of Freiburg, 79106 Freiburg, Germany; 13 Department of Neurosurgery, Alfried Krupp Hospital, 45131 Essen, Germany; 14 Department of Neurosurgery, University of Mainz, 55131 Mainz, Germany; 15 Department of Neurosurgery, Ludwig Maximilians University Hospital, 81377 München, Germany; 16 Department of Neurosurgery, Schwarzwald Baar Klinikum, 78052 Villingen- Schwenningen, Germany; 17 Institute for Epidemiology and Medical Biometry, University of Ulm, 89075 Ulm, Germany

**Keywords:** Low-grade glioma, Adjuvant therapy, Watchful waiting, Radiotherapy, Chemotherapy

## Abstract

**Purpose:**

Recent evidence-based guidelines recommend adjuvant therapy following surgery for most patients with WHO grade 2 and 3 gliomas. However, deviations from these recommendations are frequently observed in clinical practice. This study aimed to evaluate patterns of postoperative management across Germany, using multicentre registry data from certified neuro-oncology centres.

**Methods:**

We analysed data from the ongoing multicentre registry study, which prospectively collects adult patients with IDH-mutant WHO grade 2 and 3 diffuse gliomas. Patients treated at 14 certified neuro-oncology centres were included. Multivariate logistic regression was used to identify factors associated with observation, chemotherapy, or radiotherapy. We assessed concordance between guideline recommendations and actual treatment during the first year after surgery.

**Results:**

A total of 217 patients with astrocytoma or oligodendroglioma were included, of whom 169 (78%) had WHO grade 2 tumours. Observation alone was selected in 90 (53%) patients with grade 2 tumours. Gross total resection was independently associated with observation (OR 0.10; 95% CI, 0.04–0.22; *p* < 0.001). In patients aged ≥ 40 years, adjuvant treatment decisions deviated from current guidelines (OR 3.15; 95% CI, 1.70–5.95; *p* = 0.001), although age itself was not an independent predictor of treatment choice in multivariate models.

**Conclusion:**

The presence of residual tumour after surgery was the principal determinant of postoperative management in patients with WHO grade 2 gliomas. Age ≥ 40 years did not independently influence clinical decision-making. These findings highlight a gap between guidelines and real-world practice and underscore the need for more flexible, individualised treatment frameworks.

**Supplementary Information:**

The online version contains supplementary material available at 10.1007/s11060-025-05279-4.

## Introduction

IDH-mutant diffuse lower-grade gliomas (LGGs) have a significant risk of progression and recurrence and are prone to malignant transformation [1, 2]. Treatment includes surgical resection with the highest level of safety as a first-line therapy [3, 4]. Adjuvant therapy is recommended for high-risk patients, defined by one of the following risk factors: World Health Organization (WHO) grade 3 tumour, age 40 years or older, preoperative neurological deficits and incomplete resection [5, 6]. The current standard of adjuvant therapy is radiation therapy (RT) with adjuvant chemotherapy (CT) using either temozolomide (TMZ) or procarbazine and lomustine (PC) [7–9] New therapeutic strategies, including targeted therapies, are promising [10]. The therapeutic approach to LGGs is multifaceted and aims to balance maximum tumour control with potential treatment-related neurotoxicity, particularly given the often prolonged survival of patients [11].

The risk-benefit ratio of adjuvant therapy is still controversial, especially for grade 2 gliomas [12]. Following the RTOG 9802 trial, there has been a trend towards a more aggressive adjuvant management of these lesions [13]. The results of the CATNON trial support extended use of TMZ for 12 cycles in grade 3 astrocytoma [14]. In clinical practice, discrepancies may arise between guideline-based recommendations, healthcare providers and patients, as both patients and local caregivers may face challenges in adhering to these guidelines. These difficulties can have a significant impact on patients’ personal and professional lives and influence individual decision making. As targeted therapies emerge, it is important to re-evaluate existing adjuvant therapies. The aim of this study was to outline the protocols used in adjuvant therapy and to examine the factors currently influencing adjuvant treatment recommendations by analysing a German patient cohort within the multicentre prospective LoG-Glio registry.

## Methods

### Data collection and patient selection

We retrieved patients from the German prospective observational *Multicentric Registry Study on Epidemiological and Biological Disease Profile as well as Clinical Outcome in Patients with Low Grade Gliomas* (The LoG-Glio-Project, NCT02686229). Detailed inclusion and exclusion criteria and methods of data collection have been previously reported [15]. In brief, the registry enrolls all adult patients with a suspected low-grade glioma who have given informed consent. This study was performed in line with the principles of the Declaration of Helsinki. Approval was granted by the ethics committee of the University of Ulm (22. July 2015/ #201/15). The registry database was searched for patients with a history of open surgery or biopsy and confirmed IDH1/2 mutated LGG and with a follow-up length of more than three months after surgery. The final diagnosis was made based on the molecular data. If the registry dataset did not include data on the 1p/19q codeletion status, the final integrated classification provided by the respective department was used for further analysis. We extracted demographic and clinical data, final interdisciplinary adjuvant treatment recommendations, final adjuvant treatment protocols and functional scores, namely the Eastern Cooperative Oncology Group (ECOG) performance status scale and the National Institutes of Health Stroke Scale (NIHSS). We defined a preoperative neurological deficit as an NIHSS score of one or more. Patients underwent preoperative and postoperative magnetic resonance imaging (MRI) with high resolution gadolinium-based contrast enhanced T1 weighted sequences, T1 sequences without contrast agent application, T2/fluid-attenuated inversion recovery (FLAIR) sequences, diffusion weighted imaging and susceptibility weighted imaging. Residual tumour status was determined using both the surgeon’s intraoperative assessment and postoperative MRI. In cases of discrepancy, the postoperative radiological assessment was considered definitive. Absence of residual tumour on postoperative MRI was classified as low tumour burden. Recurrence was defined according to the latest Responsive Assessment in Neuro-Oncology (RANO) criteria [16]. Younger age was defined as < 40 years. Tumours involving the motor pathways, speech areas, basal ganglia, visual pathways, or brainstem were classified as eloquent. Tumour location was determined from preoperative MRI based on the treating neurosurgeon’s assessment. Patients who experienced a single recurrence following initial treatment were categorised as having a secondary lesion. If a lesion recurred following repeated treatment, it was referred to as a tertiary lesion. Missing data were obtained by contacting participating centres by e-mail or through structured monitoring visits. Cases with missing source data in the relevant time frame were excluded.

### Statistical analysis

We calculated absolute and relative frequencies for all categorical parameters and arithmetic mean, standard deviation, median and quartiles for all metrically scaled parameters. If possible, we performed a McNemar-Bowker test and calculated Cramér’s V as well as Spearman’s rho to see if either the decisions made by the multidisciplinary committee after obtaining molecular information following the initial surgery, or guideline recommendations, which were in place at the time of treatment, correlated with the treatment administered. It was not possible to calculate Cramér’s V to analyse deviations from guidelines, because guidelines recommend not only chemotherapy or radiotherapy, but also sequential therapy. Patients with secondary or tertiary lesions and who were treated before the European guidelines were published in May, 2017 were excluded [17]. We formed an additional variable for deviance of multidisciplinary board decisions and guidelines. We set up an ordinal scale by escalation of therapy (wait and scan, RT or CT, sequential therapy (RT followed by CT) and combined therapy (RT&CT followed by CT) and calculated rank differences by recommendation and received therapy. This new variable was used as a dependent variable for univariate and multivariate logistic regression analysis.

We performed univariate logistic regression analysis with adjuvant therapy as a dichotomous outcome to identify factors associated with observation. Predictor variables with a significant association (*p* < 0.1) were then used for a multivariate logistic regression analysis. The Eastern Cooperative Oncology Group (ECOG) performance status was used as a dichotomous outcome for further univariate logistic regression analysis. An ECOG score of 0–1 was considered favourable, while a score of 2–4 was considered unfavourable. Univariate and multivariate logistic regression models were also performed with chemotherapy and radiotherapy alone as dichotomous variables to assess associated factors.

We used the presence of a rank difference as a dichotomous outcome for a binary univariate logistic regression analysis to analyse factors associated with deviation from the recommendation. We used the preoperative and postoperative NIHSS, the difference of the preoperative and postoperative (delta) NIHSS, postoperative ECOG performance status, the presence of a postoperative neurological deficit, WHO grade, recurrence of the tumour, previous treatment, age, age less than 40 years old, histological and molecular findings of astrocytoma or oligodendroglioma, presence of residual tumour, eloquent site, treatment centre and time from diagnosis to treatment as independent variables.

An exploratory type 1 error level of 5% was used in all analyses to assess statistical significance. Model results were presented as odds ratios (OR) accompanied by 95% confidence intervals (CI).

All descriptive analyses were performed by SPSS V29. Logistic regression analysis, McNemar-Bowker test and the calculation of Cramér’s V and Spearman’s rho were performed by the R software for statistical computing (V4.3.2). The figures were created using R software and RAWGraphs 2.0 (rawgraphs.io).

## Results

### Patient cohort

**Fig. 1:**
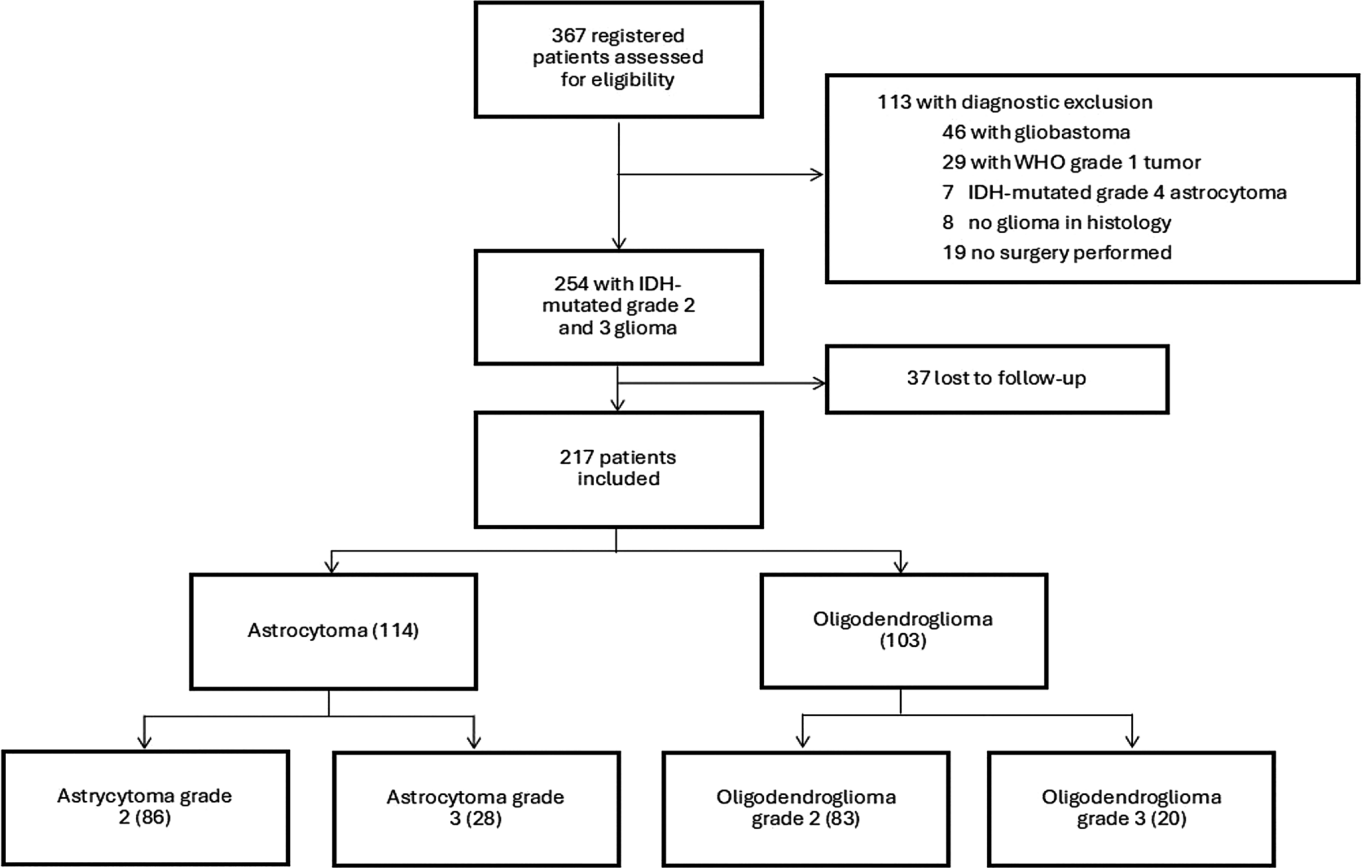
Trial profile

We included 217 of 367 patients from 14 of the 16 participating centres from November 2015 to September 2024. 150 patients were excluded from further analysis due to missing surgery, missing IDH mutation, missing follow-up, or diagnosis inconsistent with grade 2 or 3 astrocytoma or oligodendroglioma (Fig. 1). All patients had a mutation in either the IDH1 or the IDH2 isoform. The 1p19q molecular status was determined in 177 (82%) patients. Of the 40 patients for whom the 1p19q molecular status was missing, 13 (6%) had no evidence of ATRX nuclear loss. Homozygous deletion of CDKN2A/B was tested in 70 (31%) patients and was retained in all of them.

The median follow-up was 15 months (7.3–35 IQR). The median time from initial radiological diagnosis to surgical treatment or biopsy was 1 month (0.4–3.1 IQR).

Patient demographic and clinical data, tumour and treatment characteristics are summarised in Table 1. Of the patients with newly diagnosed low-grade gliomas, 111 (60%) had a history of epilepsy before diagnosis. 31 (17%) presented with headache, 10 (5%) patients had a focal neurological deficit, and 23 (12%) patients had incidental findings. In 13 (6%) cases, the reason for the first presentation was missing.

The distribution of tumour location is shown in Supplementary Table 1. The majority of the patients had a high level of functioning in terms of their ability to care for themselves, with no or mild neurological deficits (Supplementary Table 2).

**Table 1:** Patient characteristics

		patients (*n* = 217)
Age, mean (SD)		38.8 (11.7)
Gender, n (%)		
	Female Male	99 (46) 118 (54)
Glioma subtype, n (%)		
	Astrocytoma grade 2 Astrocytoma grade 3 Oligodendroglioma grade 2 Oligodendroglioma grade 3	86 (40) 28 (13) 83 (38) 20 (10)
Risk factors, n (%)		
Recurrence	Residual tumour Age ≥ 40 years Preoperative neurological deficit Primary lesion Secondary lesion Tertiary lesion	98 (45) 95 (44) 33 (15) 185 (85) 30 (14) 2 (1)
Adjuvant treatment modality, n (%)		
	No adjuvant treatment (observation) Adjuvant treatment Radiotherapy Chemotherapy Combined chemoradiotherapy Sequential therapy	93 (43) 124 (57) 10 (5) 13 (6) 35 (16) 66 (30)

### Current treatment landscape

Less than half of the patients were observed, while the remaining patients received at least one adjuvant treatment. Except for 3 (6%) patients, all patients with grade 3 glioma received adjuvant treatment. 49 (57%) of the patients diagnosed with grade 2 astrocytoma and 41 (49%) of patients diagnosed with grade 2 oligodendroglioma were observed. 114 (53%) of the patients received chemotherapy. The majority of patients with a diagnosis of astrocytoma who had chemotherapy received temozolomide. The majority of patients diagnosed with oligodendroglioma who were treated with chemotherapy received procarbazine and lomustine. Table 2 shows the different chemotherapy regimens. Of all patients who received adjuvant therapy, 14 (33%) patients with grade 2 oligodendroglioma received temozolomide. 4 (22%) patients diagnosed with grade 3 oligodendroglioma received temozolomide and 2 (11%) patients received a combination of lomustine and temozolomide. 10 (8%) patients received radiotherapy without chemotherapy, either sequential or combined, and 13 (11%) patients received chemotherapy without radiation therapy.

**Table 2:** Adjuvant therapy regimens

Adjuvant Treatment	All (124)	Astrocytoma grade 2 (37)	Astrocytoma grade 3 (27)	Oligodendroglioma grade 2 (42)	Oligodendroglioma grade 3 (18)
Radiation + Chemotherapy Temozolomide PC Lomustine	101 (81%) 67 (54%) 31 (25%) 2 (2%)	31 (84%) 30 (81%) 1 (3%) 0	26 (96%) 25 (93%) 0 0	30 (71%) 7 (17%) 21 (50%) 2 (5%)	14 (78%) 5 (28%) 9 (50%) 0
Radiation only	10 (8%)	4 (11%)	1 (4%)	4 (10%)	1 (6%)
Chemotherapy only Temozolomide PC CeTeG Other	13 (11%) 8 (6%) 1 (1%) 2 (2%) 2 (2%)	2 (5%) 1 (3%) 1 (3%) 0 0	0 0 0 0 0	8 (19%) 7 (17%) 0 0 1 (2%)	3 (17%) 0 0 2 (11%) 1 (6%)

PC: Procarbazine-lomustine

CeTeG: Lomustine-temozolomide

### Predictors of observation, radiotherapy and chemotherapy

**Fig. 2:**
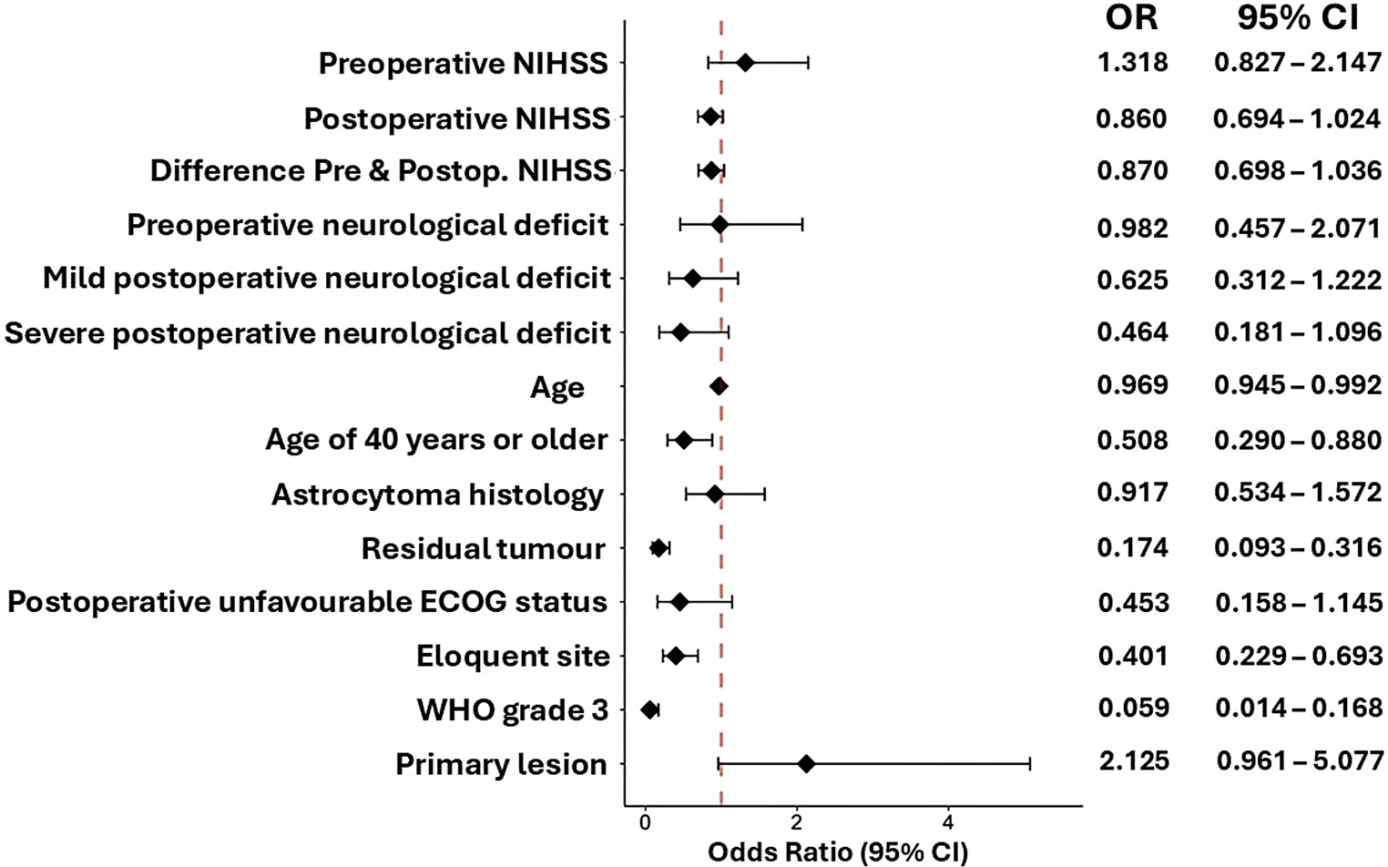
Predictive factors for observation after surgical therapy in univariate logistic regression analysis. NIHSS: National institutes of health stroke scale, ECOG: Eastern cooperative oncology group performance status, favourable: ECOG 0-1, unfavourable: ECOG 2-4, CI: Confidence Interval

Univariate logistic regression analysis showed that lower WHO grade (OR 0.06, 95% CI 0.01–1.17, *p* < 0.001), low tumour burden, meaning no residual tumour was detectable in postoperative imaging (OR 0.17, 95% CI 0.09–0.32, *p* < 0.001), younger age (OR 0.97, 95%CI 0.95–0.99, *p* = 0.008), non-eloquent site (OR 0.4 95% CI 0.23–0.69, *p* = 0.001) and primary lesions (OR 2.13, 95% CI 0.96–5.01, *p* = 0.063) were predictors of observation and inversely correlated with adjuvant therapy. Clinical performance scales which predicted observation were a lower postoperative NIHSS (OR 0.86, 95% CI 0.69–1.02, *p* = 0.094), absence of a severe postoperative neurological deficit (OR 0.46, 95% CI 0.18–1.1, *p* = 0.091) and postoperative favourable ECOG (OR 0.45, 95% CI 0.16–1.15, *p* = 0.096). Oligodendroglioma histology was not associated with adjuvant treatment strategy (OR 0.92, 95% CI 0.53–1.57, *p* = 0.75) (Fig. 2).

Further multivariate logistic regression showed that lower WHO grade (OR 0.01, 95%CI 0.01–0.07, *p* < 0.001), low tumour burden (OR 0.1, 95% CI 0.04–0.25, *p* < 0.001), younger age (OR 0.39, 95% CI 0.17–0.89, *p* = 0.026) and primary LGGs that have not recurred up to that point in time (OR 3.59, 95% CI 1.12–12.72, *p* = 0.037) were independently associated with observation strategy.

After excluding grade 3 lesions and recurrent lesions, univariate logistic regression showed that low tumour burden (OR 0.08 95% CI 0.03–0.17, *p* < 0.001), non-eloquent tumour site (OR 0.31, 95% CI 0.15–0.62, *p* = 0.01) and younger age (OR 0.97, 95% CI 0.95-1.00) were predictors of observation. Favourable postoperative ECOG (OR 0.34, 95% CI 0.10–1.02, *p* = 0.054) indicated a trend towards observation. Observation of non-recurrent grade 2 glioma was associated with low tumour burden in multivariate logistic regression (OR 0.1, 95% CI 0.04–0.22, *p* < 0.001).

Next, factors associated with either RT or CT were analysed. Univariate logistic regression analysis showed that residual tumour (OR 3.77, 95% CI 2.16–6.72, *p* < 0.001), postoperative NIHSS (OR 1.21, 95% CI 1.02–1.51, *p* = 0.031), presence of a mild or severe postoperative neurological deficit (OR 2.24, 95% CI 1.15–4.50, *p* = 0.02; OR 2.54, 95% CI 1.09–6.23, *p* = 0.035), age (OR 1.04, 95% CI 1.01–1.06, *p* = 0.003), eloquent location (OR 2.15, 95% CI 1.25–3.73, *p* = 0.006), postoperative unfavourable ECOG (OR 2.93, 95% CI 1.16–8.41, *p* = 0.022), and WHO grade 3 (OR 10.12, 95% CI 4.38–27.77, *p* < 0.001) were associated with RT (Supplementary Fig. 1).

Factors associated with CT were residual tumour (OR 4.36, 95% CI 2.47–7.86, *p* < 0.001), eloquent location (OR 2.38, 95% CI 1.38–4.15, *p* = 0.002), and WHO grade 3 (OR 11.87, 95% CI 4.88–35.64, *p* < 0.001) as shown in Supplementary Fig. 2.

Multivariate logistic regression showed that residual tumour was the only independent predictor of radiotherapy (OR 7.95, 95% CI 3.31–20.67, *p* < 0.001) and the only independent predictor of chemotherapy (OR 10.4, 95% CI 4.66–24.75, *p* < 0.001) in patients with non-recurrent grade 2 glioma.

### Adhering to guidelines and multidisciplinary neuro-oncology tumour board review recommendations

An ordinal scale was created by escalation of therapy (wait and scan, RT or CT, sequential therapy (RT followed by CT) and combined therapy (RT&CT followed by CT) and rank differences calculated by recommendation and therapy received. Postoperative multidisciplinary neuro-oncology recommendations were available for 200 of the 217 patients. There was a strong correlation of multidisciplinary neuro-oncology recommendations and therapy received (rho = 0.73, *p* < 0.001; Cramér’s V = 0.567).

Pre- and post-operative demographic data, clinical status and presence of residual tumour were available for 176 of 185 patients with primary, meaning non-recurrent LGG, who were enrolled in the study after the publication of the 2017 European guidelines for the treatment of diffuse gliomas and the 2021 updated version. A moderate correlation was observed between guideline recommendations and the therapy administered (ρ = 0.54, *p* < 0.001). Similarly, a moderate correlation existed between formal guidelines and the multidisciplinary neuro-oncology tumour board review recommendations provided to patients (ρ = 0.58, *p* < 0.001).

We conducted an analysis of factors associated with deviations from these recommendations, adjusting for the presence of grade 3 lesions. The presence of residual tumour and histopathological or molecular features consistent with astrocytoma were significantly associated with discrepancies between the multidisciplinary neuro-oncology recommendations and the treatment ultimately administered (OR = 2.81, 95%CI: 1.47–5.55, *p* = 0.002; OR = 1.93, 95%CI: 1.02–3.71, *p* = 0.045, respectively). In 6% of cases, patients who were recommended combined chemoradiotherapy instead received sequential treatment.

**Fig. 3:**
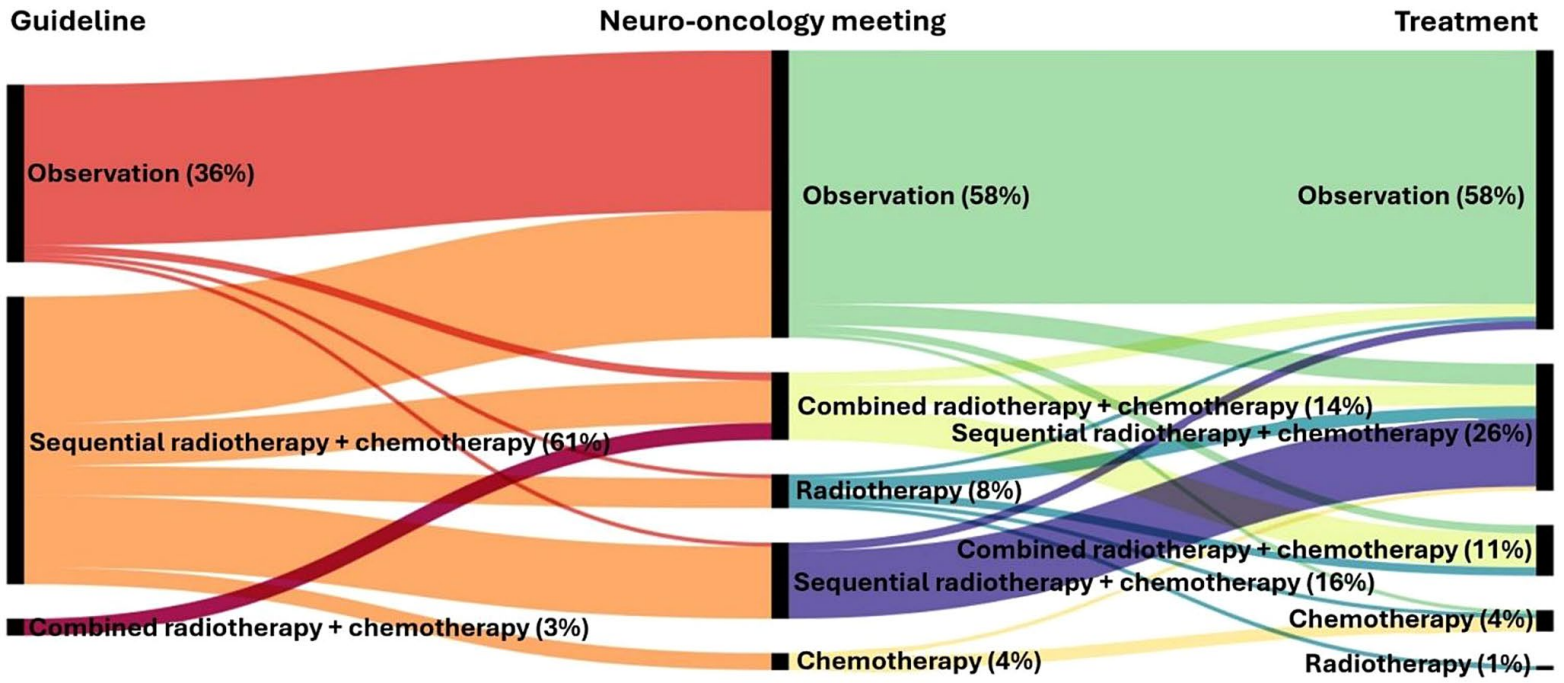
Alluvial flow diagram illustrating the interactions between the recommendations and the treatment given, and the guideline recommendation that applies in each situation for patients with non-recurrent grade 2 glioma

Age, categorised as below 40 years versus 40 years or older, was identified as a significant factor influencing deviations from guideline-based treatment (OR = 3.15, 95%CI: 1.70–5.95, *p* = 0.001). Figure 3 illustrates the interplay between treatment recommendations, the actual treatment administered, and the corresponding guideline recommendations relevant to each scenario.

## Discussion

In this study, we investigated factors influencing the decision to either observe patients with low-grade glioma or initiate adjuvant therapy with radiotherapy and chemotherapy. Low tumour burden, specifically the absence of residual tumour following surgery, emerged as an independent predictor for adopting an observation strategy. Notably, this association was significant regardless of patient age. Consequently, even patients aged 40 years or older, who would typically meet criteria for adjuvant treatment, often did not receive it if no residual tumour was detected. In contrast, the presence of residual tumour was an independent predictor for the administration of both RT and CT.

Frequent discrepancies were observed between guideline recommendations, multidisciplinary neuro-oncology meeting decisions, and the actual post-surgical treatment or observation patients received. Residual tumour and a histological or molecular diagnosis consistent with astrocytoma were associated with deviations from the multidisciplinary team’s recommendations, while patient age was associated with deviations from established clinical guidelines.

We used a prospective, multicentre patient database comprising a large and diverse cohort of individuals. This cohort reflects a representative distribution of demographic characteristics, histopathological subtypes, molecular profiles and relevant risk factors [18, 19]. This study provides real-world evidence on the post-operative management of LGG patients beyond the standardised frameworks imposed by randomised controlled trials.

The strategy of targeting only non-resectable residual tumour with adjuvant treatment is gaining wider acceptance, alongside an increasing emphasis on achieving gross total or even supramaximal resection whenever feasible [20]. Notably, long-term outcome data from the Montpellier group compellingly demonstrate that long-term survival is possible after supramaximal resection without adjuvant treatment [21]. In our cohort, which reflects a broad and representative sample of the German neuro-oncological landscape, age does not appear to be applied as an absolute criterion in clinical decision-making. This is despite recommendations in both European and national guidelines that suggest age should be a key consideration. For more than two decades, patient age and astrocytoma histology have been regarded as key factors in the risk stratification of LGG [2]. Clinical trials have consistently incorporated age, in particular, as a defining criterion for high-risk LGG [22, 23]. This is despite the fact that the cut-off point of ≥ 40 raised by Pignatti et al. was based on the median age of their cohort. Furthermore, the choice of chemotherapy has been shown to influence survival outcomes, particularly in 1p/19q-codeleted grade 3 tumours, as demonstrated in a recent French national cohort study [24]. However, more recent evidence challenges the role of age and histology grade in patients with astrocytoma as independent prognostic variables, raising questions about the true clinical significance of these factors [25]. This ongoing debate may explain why, in the absence of residual tumour in grade 2 lesions, decisions regarding adjuvant therapy versus observation are often driven more by neuro-oncologists’ attitudes or patients’ preferences than by robust prognostic markers. Whether this results in overtreatment or undertreatment remains uncertain and individualised, case-by-case management therefore continues to dominate current practice across many institutions.

Despite the known risk factors for early tumour progression, recommendations for adjuvant therapy remain controversial. In a recent Australian multicentre registry analysis, Gately et al. showed a lower treatment rate than in our study [19]. In particular, patients were less likely to receive sequential radiotherapy and chemotherapy, although the proportion of high-risk patients was higher. One reason for the cautious and restrained use of adjuvant therapy is the equivocal knowledge about the long-term cognitive effects of radiotherapy and chemotherapy in patients with diffuse glioma [26–28]. The proportion of patients receiving sequential chemoradiotherapy in this study was comparable to other European population-based cohorts, but still not all high-risk patients received adjuvant treatment [29]. The potential long-term toxicity of adjuvant treatment, including neurocognitive decline, endocrine dysfunction, and secondary malignancies, is a major concern, especially in younger patients with extended survival horizons [30, 31]. The current evidence base remains insufficient to definitively guide critical decisions such as deferring radiotherapy or opting for chemotherapy alone in specific clinical scenarios. This variability underscores a central dilemma in the counselling and management of patients with lower-grade gliomas, particularly younger individuals with IDH-mutant tumours.

Our study revealed some discrepancies with current European guidelines regarding the influence of preoperative clinical performance status on treatment decisions. While these guidelines advocate for adjuvant therapy in the presence of preoperative neurological deficits, our analysis did not identify an independent association between neurological status or overall performance score and the administration of adjuvant treatment. This finding is particularly noteworthy given that patients with larger tumours, who are more likely to exhibit tumour-related neurological symptoms, tend to have lower rates of gross total resection and are more frequently considered for adjuvant radiotherapy targeting residual disease [32]. Following this line of reasoning, it is surprising that the clinical condition was not independently associated with the adjuvant therapy.

Time from diagnosis to treatment was also not associated with the postoperative treatment or observation strategy. The optimal timing of adjuvant therapy remains a matter of debate. While early adjuvant treatment may improve tumour control, it could exacerbate long-term toxicity.

Treatment recommendations from specialist multidisciplinary neuro-oncology panels showed a moderate correlation with the patient management, but the analysis suggests a significant gap from perfect agreement. The definition of high-risk patients is slightly arbitrary and has relevant variation in multiple guidelines resulting in different recommendations for treatment or observation [33]. Adjuvant treatment or observation is provided by a variety of healthcare professionals, complicating this challenge. In some cases, patients may be treated or observed by radiotherapists or oncologists who are not familiar with the rare entity of LGG. Balancing these recommendations with personal goals and expectations can be a challenging endeavour. Patients may also be reluctant to undergo adjuvant treatment because they fear it will reduce their quality of life. Current guidelines only partially address this.

Another reason for the discrepancy between panel decisions and actual treatment is that a small proportion of panel decisions in recent years have included a recommendation for combined chemoradiotherapy for patients with astrocytoma. The 2021 EANO guidelines no longer recommend combined therapy for patients with grade 2 and 3 astrocytoma, only sequential therapy. As the proportion is small, it does not explain the whole discrepancy.

### Limitations

Although the database used in this study was prospectively established, the analysis itself was conducted retrospectively. This inherently limits the ability to fully capture and analyse all factors influencing clinical decision-making, which is a recognised constraint of registry-based research. Individual treatment decisions may have been guided by clinical nuances not documented in the registry. 3 centres contributed 54% of the total patient cohort, introducing the possibility of centre-specific treatment preferences. When centres exhibit a strong preference for a specific treatment approach, isolating causal effects becomes more challenging. Nevertheless, the large number of participating centres, each contributing multiple neuro-oncology specialists, makes it unlikely that treatment decisions were driven solely by individual familiarity. This reduces the likelihood that our results were substantially confounded by centre-specific or individual practice patterns. The registry case report form used does not indicate whether vincristine was omitted in patients who received procarbazine and lomustine (PC regimen). Based on information of all participating centres, which had uniformly discontinued the use of vincristine at the onset of the study, we assumed that vincristine was not administered.

The relatively short observation period in our cohort probably encompasses all immediate postoperative management decisions, however, long-term follow-up is essential to identify late treatment-related toxicities that may remain undetected in short-term analyses. Assessing outcomes that truly reflect the long-term burden of disease requires extended observation over many years. The present study is limited to evaluating short-term decision-making, without the ability to assess the broader, patient-centred impact of these decisions over time. This short follow-up window may have led to an underestimation of delayed adjuvant treatment, particularly for patients initiated on therapy beyond three months post-surgery. Nonetheless, most clinical guidelines currently recommend initiating adjuvant therapy within 12 weeks, suggesting that the majority of patients requiring chemotherapy would have begun treatment within the timeframe captured [34]. In our cohort, 3 patients who received radiotherapy alone had a short follow-up of 3 to 4 months. Given the small proportion, it is unlikely that this study missed a significant number of patients who received delayed chemotherapy. During the extended study period, several updates to European, American and national treatment guidelines were published. The study does not assess the latency between guideline publication and their adoption in clinical practice, which remains a relevant and yet unquantified variable. Another limitation is the absence of quantitative measurement of residual tumour. Volumetric analysis was not performed, and therefore subtle remnants, potentially influencing treatment decisions, may have gone unrecognised in the dataset. Even small residual tumour volumes may prompt consideration of adjuvant therapy, with the rationale of reducing recurrence risk and delaying malignant transformation. This approach is further supported by prognostic evidence indicating that the extent of resection is among the strongest predictors of outcome in patients with low-grade glioma. Conversely, surveillance of minimal residual disease may spare patients from the potential toxicities of adjuvant treatment, particularly given that small remnants can remain stable for years. In such cases, close monitoring with initiation of therapy at progression may represent a reasonable alternative. Importantly, our data do not permit evaluation of the fundamental question of whether very early treatment of minimal residual disease improves outcomes in this patient population, nor do they capture how this clinical scenario is currently managed in real-world practice.

## Conclusions

This study provides evidence that the presence of residual tumour is a principal determinant in the postoperative management of patients with lower-grade glioma. Future research should aim to clarify whether residual tumour *volume*, in addition to its mere presence, plays a significant role in clinical decision-making, and to what extent age functions as an independent prognostic factor.

Our findings also indicate that established clinical guidelines and multidisciplinary recommendations are not consistently implemented in real-world practice. Further investigation, particularly incorporating the patient perspective, is warranted to better understand the underlying reasons for these deviations. These insights may ultimately inform revisions of both European and national guidelines, fostering a more flexible framework that accounts for the complexities and individualised nature of treatment decision-making in LGG management.

## Supplementary Information

Below is the link to the electronic supplementary material.

Supplementary Material 1

## Data Availability

The datasets analysed during the study are available from the corresponding author on reasonable request.
