# Supplementary material for: Low tumour burden is associated with observation after surgery in patients with grade 2 astrocytoma and oligodendroglioma: results from the prospective multicentre LoG-Glio registry
Source: J Neurooncol. 2025 Oct 15;176(1):14. doi: 10.1007/s11060-025-05279-4 (PMC12528241; doi:10.1007/s11060-025-05279-4)
Supplement: Supplementary file 1 — Supplementary Material 1 [file 11060_2025_5279_MOESM1_ESM.pdf]

Supplementary Table 1. Tumour location.

		patients (n=217)
**Location, n (%)**		
	**frontal**	141 (65)
	**parietal**	24 (11)
	**temporal**	33 (15)
	**occipital**	3 (1)
	**insular**	15 (7)
	**basal ganglia**	1 (1)
**non-eloquent site, n (%)**		96 (44)
**eloquent site, n (%)**		
	motor	68 (31)
	speech	31 (14)
	basal ganglia	7 (3)
	visual cortex	8 (4)
	other	7 (3)
**hemisphere, n (%)**		
	left	98 (45)
	right	117 (54)
	both	2 (1)

Supplementary Table 2. Functional outcome

A

ECOG	Preop	Postop	6 Mo	12 Mo
**0**	79.7%	55.8%	63%	74%
**1**	17.9%	33.2%	33%	22.2%
**2**	1.4%	9.5%	4%	3.8%
**3**	0.5%	1%	0%	0%
**4**	0.5%	0.5%	0%	0%
**Overall**	212	208	121	81

ECOG: Eastern Cooperative Oncology Group performance status

B

Neurological deficit	Postop	6 Mo	12 Mo
**none**	65%	87%	93.2%
**mild***	22.6%	10%	5.4%
**severe****	12.4%	3%	1.4%
**overall**	217	119	74

*****Slight new neurological deficit: deterioration by one degree of strength or accentuation of aphasia (max. 1 point National Institutes of Health Stroke Scale)*

***Severe new neurological deficit: for significant new deficits > 1 degree of strength or significant aphasia (more than 1 point on National Institutes of Health Stroke Scale)*

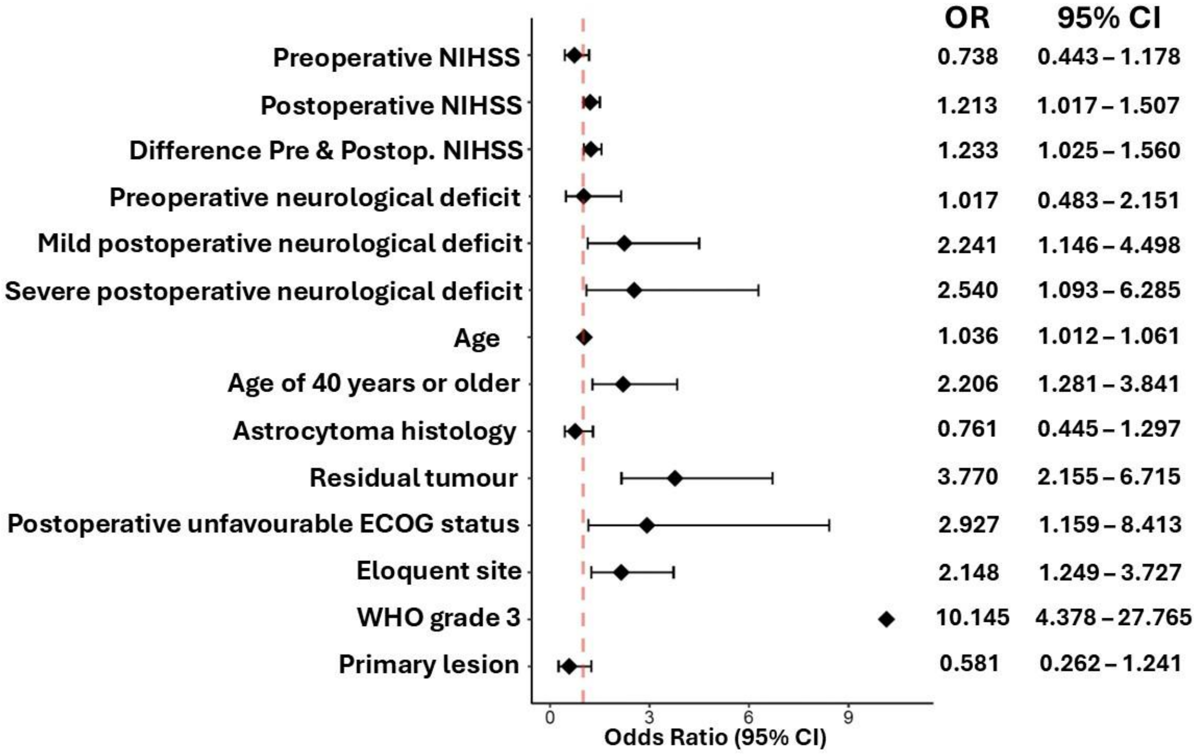

Supplementary Figure 1. Predictive factors for adjuvant radiotherapy after surgical therapy in univariate logistic regression analysis.

NIHSS: National Institutes of Health Stroke Scale

ECOG: Eastern Cooperative Oncology Group performance status

favourable: ECOG 0-1

unfavourable: ECOG 2-4

CI: Confidence Interval

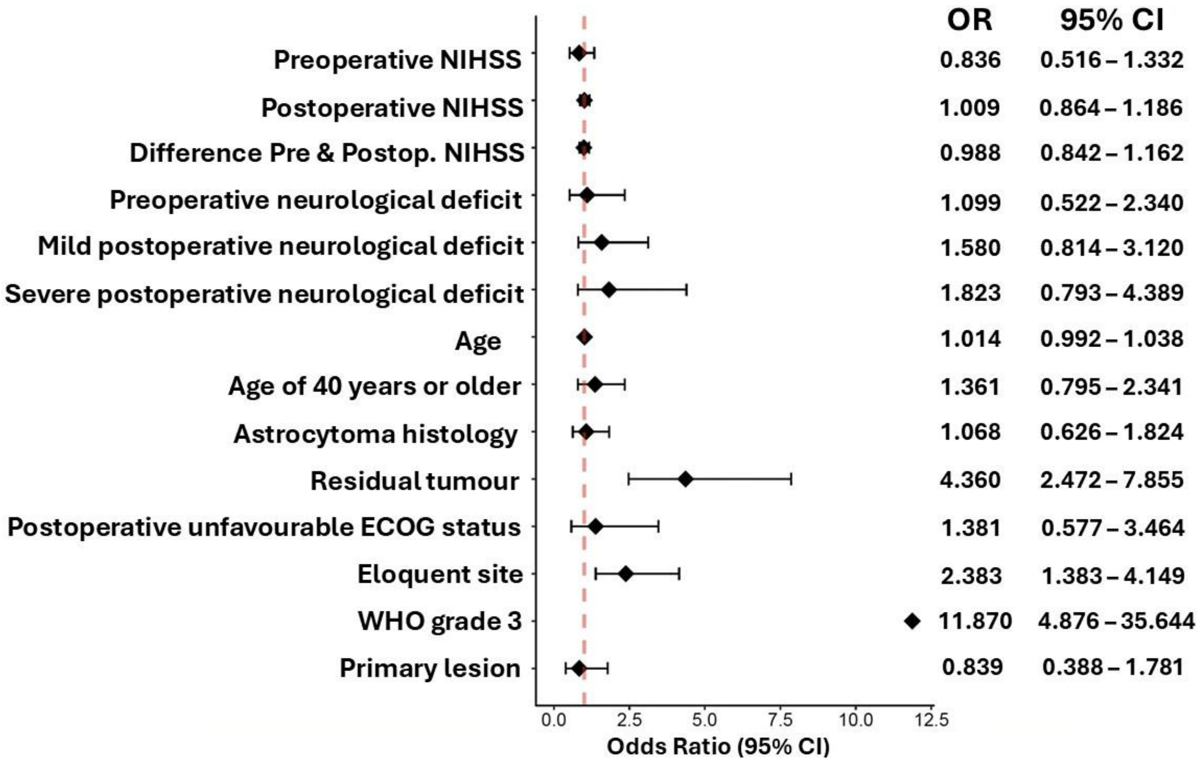

Supplementary Figure 2. Predictive factors for adjuvant chemotherapy after surgical therapy in univariate logistic regression analysis.

NIHSS: National Institutes of Health Stroke Scale

ECOG: Eastern Cooperative Oncology Group performance status

favourable: ECOG 0-1

unfavourable: ECOG 2-4

CI: Confidence Interval
